# Oral Inflammation and Human Papilloma Virus Association among Hispanics

**DOI:** 10.1155/2023/7247976

**Published:** 2023-12-11

**Authors:** Maira A. Castañeda-Avila, Cynthia M. Pérez, José Vivaldi, Elba C. Díaz-Toro, Jeslie M. Ramos-Cartagena, Oelisoa M. Andriankaja, Ana P. Ortiz

**Affiliations:** ^1^Department of Population and Quantitative Health Sciences, University of Massachusetts Chan Medical School, Worcester, Massachusetts, USA; ^2^Department of Biostatistics and Epidemiology, Graduate School of Public Health, University of Puerto Rico Medical Sciences Campus, San Juan, Puerto Rico, USA; ^3^School of Dental Medicine, Ana G. Méndez University, San Juan, Puerto Rico, USA; ^4^Department of Restorative Sciences, School of Dental Medicine, University of Puerto Rico, San Juan, Puerto Rico, USA; ^5^Hospital of the University of Puerto Rico Comprehensive Cancer Center, San Juan, Puerto Rico, USA; ^6^Division of Cancer Control and Population Sciences, University of Puerto Rico Comprehensive Cancer Center, San Juan, Puerto Rico, USA; ^7^Center for Clinical Research and Health Promotion, School of Dental Medicine, University of Puerto Rico Medical Sciences Campus, San Juan, Puerto Rico, USA

## Abstract

**Background:**

Identifying factors related to persistent human papillomavirus (HPV) infection is essential to reduce the incidence of HPV-related cancers.

**Objective:**

To evaluate whether gingival/periodontal inflammation is associated with oral HPV infection.

**Methods:**

This cross-sectional study (*n* = 740) uses data from the follow-up visit of the San Juan Overweight Adults Longitudinal Study, which recruited overweight/obese adults aged 40–65 from Puerto Rico. Participants completed a dental examination and two interviews (face-to-face/ACASI) and provided oral rinse samples for HPV detection. Oral inflammation was assessed using two definitions: (1) the number of sites with bleeding on probing (BOP), and (2) the number of teeth with probing pocket depths (PPD) ≥ 4 mm and BOP. Multivariate logistic regression was used to assess the association between oral inflammation and oral HPV.

**Results:**

Nearly three-quarters (72%) of participants were female, and 68% had 50 years or older. Participants with HPV had a higher mean number of sites with BOP (15.5 vs. 10.1) and teeth with PPD ≥ 4 mm and BOP (8.5 vs. 3.2) than participants without HPV (*p* < 0.05). After adjusting for sex, age, income, and the number of oral sex partners, the odds of having an oral HPV infection increased by 3% (95% confidence interval (CI): 1.00–1.06) for any additional sites with BOP and 5% (95% CI: 1.02–1.09) for any other teeth with PPD ≥ 4 mm and BOP.

**Conclusions:**

We found that oral inflammation was associated with oral HPV infection among adults in Puerto Rico. Future studies need to further investigate the underlying mechanisms.

## 1. Introduction

Periodontitis is a highly prevalent chronic local inflammation that leads to the formation of pockets between the teeth and gums, destruction of the tissues supporting the teeth, and tooth loss [1, 2]. This chronic local inflammation has been associated with systemic conditions, including diabetes, cardiovascular disease, chronic obstructive pulmonary disease, and chronic kidney disease [3–6]. Nearly half of adults aged 30 years or older in the United States have periodontitis [7]. On the other hand, human papillomavirus (HPV) infection is the most prevalent sexually transmitted infection [8, 9], and it has been estimated that 70% of oropharyngeal cancers are associated with oral HPV infection [10].

Recently, a link between periodontal disease and oral HPV infection has been proposed. Although results from studies have been contradictory, it has been hypothesized that the periodontal pocket provides an opportunity for HPV acquisition and persistence in the oral mucosa and may serve as a reservoir for latent HPV [11–13]. An alternative hypothesis is that chronic tissue inflammation might contribute to the persistence of oral HPV infection [13]. The biological mechanisms of this association are not precise since research in this area has been limited.

Our previous research has documented a prevalence of 6% for oral HPV infection and 20% for severe periodontal disease in a sample of Hispanic adults in Puerto Rico, as well as a positive association between severe periodontal disease and oral HPV infection [14]. However, we were interested in evaluating other markers of periodontal disease to explore these relationships further. In this study, we assessed whether gingival and periodontal inflammation were associated with oral HPV infection, using as indicators gingival bleeding on probing (BOP) and probing pocket depth (PPD). These parameters can help us evaluate in more detail the biological mechanisms of the association between periodontal disease and oral HPV infection (and the previous hypotheses proposed), as the presence of deep pocket depth could act as a reservoir for latent HPV infection and the presence of BOP may reflect the presence of chronic oral tissue inflammation.

## 2. Materials and Methods

### 2.1. Study Design and Setting

This cross-sectional substudy was conducted among eligible participants from the “Periodontitis and oral human papillomavirus infection among Hispanic adults,” who completed the 3-year follow-up visit (2014–2016) of the “San Juan Overweight Adults Longitudinal Study (SOALS) whose baseline visit occurred from 2011 to 2013.” Using mass media approaches, SOALS recruited overweight/obese adults aged 40–65 who were residents of the San Juan metropolitan area [14, 15]. Baseline exclusion criteria included physician-diagnosed diabetes mellitus, pregnancy, and history of systemic conditions (e.g., endocarditis, congenital heart murmurs, heart valve disease, and bleeding disorders) that could increase the risk of systemic complications during the full periodontal assessment [14, 15]. The sample size of the current study was determined based on the available data from the SOALS study without a formal sample size calculation. Of the 773 participants who came to the follow-up SOALS examination visit, 771 accepted to complete the present study procedures. Participants were excluded from the current analysis if they did not complete the oral HPV collection or periodontal evaluation procedures or had unsatisfactory HPV results in the laboratory (e.g., samples negative for *β*-globin gene amplification). The analytic sample for the present study was 740 (96% of recruited participants). Given the predetermined sample size of 740 participants for the parent study, the estimated power for the association of the number of sites with BOP and oral HPV infection in this study was 67%. In comparison, the power for the association of PPD and BOP with oral HPV infection was 76%. The Institutional Review Board of the University of Puerto Rico, Medical Sciences Campus, approved this study, and all participants provided written informed consent.

### 2.2. Study Variables

#### 2.2.1. Oral HPV Infection

The primary outcome variable was oral HPV infection. A self-collected oral rinse sample was obtained from each participant using the National Health and Nutrition Examination Survey (NHANES) methodology [16]. Participants were asked to rinse/gargle with 10 mL of mouthwash (Scope) for 30s, spit the mouthwash into a 50 mL sterile collection cup, and not spill the liquid, and instructed to close the cup tightly [14]. HPV typing was performed at the University of California San Francisco HPV Virology Core Laboratory using polymerase chain reaction. Samples were typed by dot-blot hybridization using 39 type-specific probes. A sample was classified as positive for oral HPV infection if it was positive for the consensus probes or any specific HPV type probes; this included persons with high-risk, low-risk, or unknown HPV types [14].

#### 2.2.2. Gingival/Periodontal Inflammation

The primary predictor variable was gingival/periodontal inflammation. A full mouth clinical exam was performed using a modified version of the oral health component of NHANES and procedures performed by a trained dental examiner. The oral examination was assessed at six sites for all teeth, excluding the third molars, including PPD and BOP measures, using a periodontal probe inserted in the base of the sulcus or pocket. BOP was present if any buccal or lingual tooth sites bled within approximately 20s after probing. Oral inflammation was defined as (1) the number of tooth sites with BOP (indication of the presence of reversible plaque-induced gingivitis or irreversible periodontitis) and (2) the number of teeth with PPD ≥ 4 mm and BOP (indicator of the number of teeth with possible periodontitis) [17–19].

#### 2.2.3. Covariates

A face-to-face interview was used to collect sociodemographic and lifestyle characteristics, including sex (male, female), age (40–49, 50–64 years), marital status (single, divorced, widowed, married or cohabiting), years of education (≤12, >12), income (<$ 20,000, ≥$ 20,000), and smoking (never, former, current). The lifetime number of sexual partners (0–9, ≥10) and oral sexual partners (0, 1–5, ≥6) was collected through an audio computer-assisted self-interview.

### 2.3. Statistical Analysis

Student's *t*-test or analysis of variance (ANOVA) was used to evaluate the mean number of sites with BOP and teeth with PPD ≥ 4 mm and BOP across baseline characteristics. The Mann–Whitney or Kruskal–Wallis test compared the median number of sites with BOP and teeth with PPD ≥ 4 mm and BOP across baseline characteristics. The Mann–Whitney test was also used to assess the difference in the number of tooth sites with BOP and the number of teeth with PPD ≥ 4 mm and BOP by HPV status.

A multivariable binary logistic regression model was used to evaluate the association between oral inflammation and oral HPV infection. The model was adjusted by covariates significantly associated (*p* < 0.05) in the bivariate analysis with oral inflammation, HPV infection, and other relevant variables in the literature. All analyses were conducted using STATA statistical software (version 17, Stata Corporation, College Station, TX).

## 3. Results

Most study participants were female (72%), middle-aged individuals (68%), and had no history of binge drinking (85%) or smoking (64%) (Table 1). A total of 42 participants were positive for oral HPV (5.6%). Women had a significantly higher mean number of sites with BOP (12.8 vs. 9.4) and number of teeth with PPD ≥ 4 mm and BOP (5.8 vs. 2.7) compared to men (Table 2). Participants who had 12 years or less of education also had a significantly higher mean number of sites with BOP (10.9 vs. 9.7) and number of teeth with PPD ≥ 4 mm and BOP (4.2 vs. 2.7) than those with more years of education. Those who reported binge drinking and at least 10 sexual partners in their lifetime also had significantly higher mean number of sites with BOP and number of teeth with PPD ≥ 4 mm and BOP than their counterparts. There were also significant differences in the median number of sites with BOP and number of teeth with PPD ≥ 4 mm and BOP across sex, income, and lifetime number of sexual partners.

**Figure 1:**
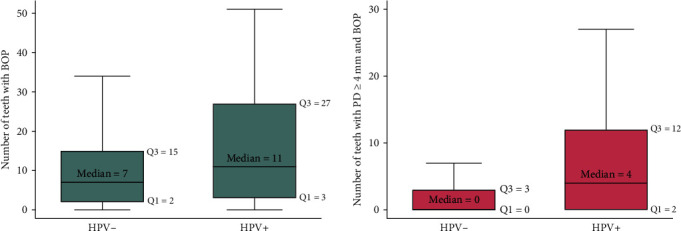
Boxplots of the number of sites with BOP and teeth with PPD ≥ 4 mm and BOP according to oral HPV infection.

Participants with oral HPV had a significantly higher median number of sites with BOP than participants without HPV (11 vs. 7, *p* − value=0.03) (Figure 1). Additionally, participants with oral HPV also had a higher mean number of teeth with both PPD ≥ 4 mm and BOP than participants without HPV (4 vs. 0, *p* − value=0.001). After adjusting for sex, age, annual income, and the number of oral sex partners, the odds of having oral HPV infection increased significantly with each unit increase in the number of sites with BOP (odds ratio (OR) = 1.03, 95% confidence interval (CI): 1.00–1.06, *p*=0.017) and the number of teeth with PPD ≥ 4 mm and BOP (OR = 1.05, 95% CI: 1.02–1.09, *p*=0.001) (Table 3).

## 4. Discussion

This study evaluated the association of two markers of oral inflammation and infection with oral HPV. In this sample of Hispanic adults, we found that the odds of oral HPV infection increased significantly as the number of sites with BOP and teeth with PPD ≥ 4 mm and BOP increased. These findings indicate that markers of oral inflammation and periodontal disease may play a role in oral HPV infections, as has been suggested in previous studies.

A recent narrative review of 12 articles evaluating the association between periodontitis and HPV concluded that results are conflicting and still inconclusive [13]. Nonetheless, several studies found an association between oral HPV infection and periodontitis. The largest differences between the studies were variations in study methodology and definitions used to assess periodontitis and oral HPV infection status [13]. A significant component of the routine periodontal examination is BOP, which is known to be a classic sign of periodontal inflammation and is highly correlated with active periodontal disease [20].

We found that the odds of having an oral HPV infection increased by 3% for any other tooth site with BOP. Few studies have evaluated the association between BOP and HPV. A previous study among Japanese women (*n* = 46) found a significant association between BOP and oral HPV-16 infection [21]. This provides further evidence supporting the notion that BOP, as an indicator of active periodontal disease, might also be a potential risk factor for oral HPV infections.

We also evaluated the number of teeth with PPD ≥ 4 mm and BOP to indicate the number of teeth with possible periodontitis. We found that the odds of having oral HPV infections increased by 5% for any other tooth with PPD ≥ 4 mm and BOP. Those findings are consistent with previous studies that found an association between periodontitis and oral HPV, particularly among participants with severe periodontitis [14]. This suggests that individuals with more advanced periodontal disease may need closer monitoring for oral HPV infections.

## 5. Conclusion

Oral inflammation, measured by the number of sites with BOP and the number of teeth with PPD ≥ 4 mm and BOP, may contribute to oral HPV infection. Evaluating specific markers of inflammation is important to provide information that ultimately will help offer preventive strategies for oral HPV-related cancers.

## Figures and Tables

**Table 1:** Sociodemographic characteristics of the study population.

Characteristics	*n* (%)
Sex	
Male	204 (27.6)
Female	536 (72.4)
Age (years)	
40–49	237 (32.0)
50–64	503 (68.0)
Marital status	
Single, divorced, widowed	408 (55.1)
Married or cohabiting	332 (44.9)
Education	
≤12 years	403 (54.5)
>12 years	337 (45.5)
Income	
<$ 20,000	396 (53.7)
≥$ 20,000	342 (46.3)
Smoking	
Never	469 (63.9)
Former	130 (17.7)
Current	135 (18.4)
Binge drinking	
No	630 (85.1)
Yes	110 (14.9)
Lifetime number of sexual partners	
0–9	518 (74.1)
≥10	181 (35.9)
Lifetime number of oral sexual partners	
0	98 (13.3)
1–5	484 (65.6)
≥6	156 (21.1)

**Table 2:** Comparison of sociodemographic characteristics of the study population by the number of teeth with BOP and number of teeth with PD ≥ 4 mm and BOP.

	Number of sites with BOP				Number of teeth with PPD ≥ 4 mm and BOP			
	Mean (SD)	*p* ^*∗*^	Median (IQR)	*p* ^*∗∗*^	Mean (SD)	*p* ^*∗*^	Median (IQR)	*p* ^*∗∗*^
Sex								
Male	9.4 (10.0)	<0.05	6 (2–15)	<0.05	2.7 (5.6)	<0.05	0 (0–2.5)	<0.05
Female	12.8 (12.6)		9 (3–19)		5.8 (9.3)		2 (0–8)	
Age (years)								
40–49	11.5 (11.0)	0.06	8 (2–17)	0.03	3.6 (6.8)	0.76	0 (0–4)	0.56
50–64	9.9 (10.7)		6 (2–15)		3.5 (7.1)		0 (0–4)	
Marital status								
Single, divorced, widowed	10.0 (10.7)	0.33	6 (2–15)	0.27	3.5 (6.8)	0.95	0 (0–4)	0.94
Married or cohabiting	10.8 (11.0)		7 (2–16)		3.5 (7.2)		0 (0–4)	
Education								
≤12 years	10.9 (11.3)	<0.05	7 (2–17)	0.33	4.2 (7.4)	<0.05	1 (0–5)	<0.05
>12 years	9.7 (10.3)		6 (2–14)		2.7 (6.4)		0 (0–3)	
Income								
<$ 20,000	11.2 (11.5)	0.81	7 (2–17)	0.05	4.3 (7.7)	<0.05	1 (0–5)	<0.05
≥$ 20,000	9.4 (9.9)		6 (2–14)		2.6 (5.8)		0 (0–3)	
Smoking								
Never	10.5 (10.7)	0.13	7 (2–15)	0.56	3.0 (6.5)	0.11	0 (0–3)	<0.05
Former	10.1 (11.0)		5.5 (2–14)		3.9 (7.0)		1 (0–5)	
Current	11.9 (11.1)		8 (2–18)		5.1 (8.3)		1 (0–6)	
Binge drinking								
No	10.1 (10.8)	<0.05	6 (2–15)	0.09	3.4 (6.9)	<0.05	0 (0–3)	0.09
Yes	11.8 (11.0)		9.5 (3–18)		4.5 (3.1)		1 (0–6)	
Lifetime number of sexual partners								
0–9	9.8 (10.5)	<0.05	6 (2–15)	<0.05	3.1 (6.6)	<0.05	0 (0–3)	<0.05
≥10	11.8 (11.6)		8 (2–18)		4.5 (7.7)		1 (0–5)	
Lifetime number of oral sexual partners								
0	8.7 (10.7)	0.17	5 (1–13)	0.12	3.8 (8.9)	0.07	0 (0–3)	0.05
1–5	10.4 (10.5)		7 (2–16)		3.1 (6.1)		0 (0–3)	
≥6	11.3 (11.9)		7 (2–17)		4.6 (8.1)		1 (0–5)	

^*∗*^*p*-Value computed from Student's *t*-test or ANOVA.  ^*∗∗*^*p*-Value computed from Mann–Whitney test or Kruskal–Wallis.

**Table 3:** Odds ratio (OR) of the associations between increased number of sites with BOP or number of teeth with PPD ≥ 4 mm and BOP and HPV status.

Oral inflammation	HPV Infection	
	Crude OR (95% CI)	Adjusted OR (95% CI) ^*∗*^
Number of sites with BOP	1.04 (1.01–1.06)	1.03 (1.00–1.06)
Number of teeth with PPD ≥ 4 mm and BOP	1.06 (1.03–1.09)	1.05 (1.02–1.09)

^*∗*^Adjusted by sex, age, income, and lifetime number of oral sex partners.

## Data Availability

The data used to support the findings of this study are restricted by the Institutional Review Board of the University of Puerto Rico Medical Science Campus in order to protect participants' privacy. Data are available from Ana P. Ortiz for researchers who meet the criteria for access to confidential data.

## Additional Points

*Strengths and Limitations*. This study has several strengths, including validated methods for oral HPV detection and typing and periodontitis and ACASI methodology to collect sensitive information related to oral HPV risk factors. In addition, we evaluated the status of gingival/periodontal inflammation instead of the periodontal disease definition of the Centers for Disease Control and Prevention/American Academy of Periodontology (CDC/AAP) or attachment loss alone, which has the limitation of not distinguishing past or present experience of periodontitis. We used two case definitions of inflammatory status, which can indicate the disease activity or inflammatory status of the gingival tissue or the periodontal pocket at the time of oral clinical evaluation. We also recognized some limitations in the dental evaluation. Even though participants completed a full mouth-validated probing pocking examination, we only recorded BOP on two sites per tooth due to the high level of bleeding at each site, making it difficult to differentiate one site contaminated from other areas in the pocket. Finally, given that this is a cross-sectional study, we cannot determine the temporal sequence between oral inflammation and oral HPV infection.
